# Profit versus Quality: The Enigma of Scientific Wellness

**DOI:** 10.3390/jpm12010034

**Published:** 2022-01-03

**Authors:** Katrina Carbonara, Adam J. MacNeil, Deborah D. O’Leary, Jens R. Coorssen

**Affiliations:** 1 Department of Health Sciences, Faculty of Applied Health Sciences, Brock University, 1812 Sir Isaac Brock Way, St. Catharines, ON L2S 3A1, Canada; kcarbonara@brocku.ca (K.C.); amacneil@brocku.ca (A.J.M.); doleary@brocku.ca (D.D.O.); 2 Brock-Niagara Centre for Health and Well-Being, 130 Lockhart Dr, St. Catharines, ON L2T 1W5, Canada; 3 Department of Biological Sciences, Faculty of Mathematics and Science, Brock University, 1812 Sir Isaac Brock Way, St. Catharines, ON L2S 3A1, Canada

**Keywords:** precision medicine, personalized medicine, functional medicine, wellness, healthspan, preventive medicine

## Abstract

The “best of both worlds” is not often the case when it comes to implementing new health models, particularly in community settings. It is often a struggle between choosing or balancing between two components: depth of research or financial profit. This has become even more apparent with the recent shift to move away from a traditionally reactive model of medicine toward a predictive/preventative one. This has given rise to many new concepts and approaches with a variety of often overlapping aims. The purpose of this perspective is to highlight the pros and cons of the numerous ventures already implementing new concepts, to varying degrees, in community settings of quite differing scales—some successful and some falling short. Scientific wellness is a complex, multifaceted concept that requires integrated experimental/analytical designs that demand both high-quality research/healthcare and significant funding. We currently see the more likely long-term success of those ventures in which any profit is largely reinvested into research efforts and health/healthspan is the primary focus.

## 1. Introduction

One size does not fit all, yet the design of the modern healthcare system is almost entirely centered on this notion. The practice of traditional Western medicine has been to identify and treat one disease or symptom with, in general, one medication for the entire population. Largely ignored is that no two individuals are identical, (epi)genetically or otherwise, and ethnic, cultural, and lifestyle differences matter—arguably, nurture being at least as important as nature, and their interaction being the real need for focus [1,2,3,4]. With the critical exception of specific monogenic disorders, in the vast majority of cases, genes are not strictly fate, although they may be predictive of potential/possible health issues. Thus, manifestations of a disease can differ drastically between individuals, as can their responses to the same treatment. Nonetheless, the current general model of medicine is mainly reactive and based on average responses, normally focusing on the identification and treatment of a disease or its symptoms once they are clinically identifiable—although, in some cases, waiting for symptoms is waiting too long [5]. While there are preventative measures within this model, these are mainly designed to limit the further development of identified health complications. While these methods can be largely successful, the general approach is still reactive in nature. More recently, there has been a shift to focus on genuine early and more broadly preventive approaches, although some will argue that this trend started with the concept of “alternative medicine” or, perhaps even more accurately, with the traditional medical practices of Asia and India. From the viewpoint of Western evidence-based medicine, this “newer” approach is centered more on underlying processes—indeed, etiology—and less on the generalized disease state per se [6].

**Figure 1:**
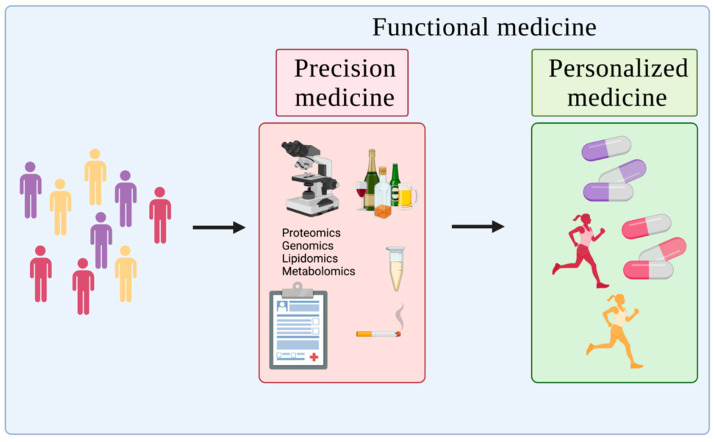
Precision vs. personalized vs. functional medicine. Precision medicine identifies differences in individuals, categorizing based on environmental, biological, and psychosocial factors. Personalized medicine takes these differences and implements preventions/treatments tailored to the individual. Functional medicine is an overarching term that seeks to encompass both precision and personalized medicine.

While this newer, preventive approach to healthcare is still in the early stages, there is a rapidly growing research platform based on these diagnostic, prognostic, and therapeutic strategies that are tailored to the needs of individual patients [7]. While somewhat broadly and generically termed precision, personalized, or functional medicine—the terms often being used interchangeably—there are important distinctions. Precision medicine is a science and involves the tailoring of treatments to the individual features of a patient based on genetic, phenotypic, and/or psychosocial characteristics [8,9]. Broadly, individuals are classified into subpopulations that differ in their susceptibility to a particular disease, in the etiology and/or prognosis of those diseases, or in their response to a specific treatment [8]. In contrast, personalized medicine is a practice that commercializes precision medicine research with the aim of matching individuals with the prevention and therapy techniques that are “best” suited to them [10,11]. Obviously, the latter comes with a plethora of assumptions (and often no small amount of salesmanship), especially since we cannot currently effectively assess the most likely future health of a given individual. In this regard, functional medicine tends to serve as somewhat of an umbrella term that encompasses precision and personalized medicine. It essentially seeks to encompass understanding of the fundamental science and the design/implementation of the practice [6] (Figure 1).

Furthermore, there is a newer term linked to precision and personalized medicine that incorporates the maintenance and improvement of health and wellness (i.e., salutogenesis [12]), and thus healthspan (i.e., that portion of an individual’s life spent in good health, free from disease and disability [13]). Scientific wellness, a term coined by Leroy Hood, is a “quantitative, data-informed approach to maintaining and improving health, as well as avoiding disease” [14,15]. Importantly, this embodies the essence of what Hood has termed P4 medicine: predictive, preventive, personalized, and participatory [14]. The main goal of scientific wellness is to create precisely personalized health strategies and treatments that improve health/prevent dysfunctions, reverse disease transition, and reduce costs [15]. As all three target medical and lifestyle interventions, the main difference is that precision/personalized medicine leans more toward medical intervention, while scientific wellness leans more toward scientifically guided lifestyle intervention. Clearly, these are complementary rather than mutually exclusive approaches. There is thus a broad and growing interest in developing and applying the concepts of scientific wellness, and numerous research-based and business endeavors seek to establish these as mainstream approaches to improving healthspan and wellness [16,17]. The overall aim is to reduce our more multifactorial and longer-term healthcare burdens by addressing them before they start or, at the very least, substantially reducing the impact of these burdens both in terms of severity and time lost. A literature review was conducted with a PubMed search, starting a year before Arivale was initiated to the present day (2014–2021), using the keywords “Arivale” and “scientific wellness”. Review sources were used to identify companies involved in such programs, and additional web-based searches were used to find accompanying websites of specific ventures. We acknowledge that this is a perspective paper, with inherent biases.

## 2. Current Strategies

### 2.1. Research-Based Ventures (Voluntary Participants)

Of the many research ventures that exist, most are working with a mandate to enhance precision medicine and scientific wellness. In no particular order, these include, firstly, the China Kadoorie Biobank (University of Oxford, Old Campus Road, Oxford OX3 7LF, UK), which was created to investigate the main genetic and environmental causes of common chronic diseases in the Chinese population. While several important causes, or at least factors, affecting the development of various chronic diseases are already known, most data are based on studies conducted in the West and do not generally take into account the large geographic and ethnic differences in disease rates around the world. Thus, the main objective of the Kadoorie Biobank is to assess the effects of established and emerging risk factors for multiple diseases across a variety of circumstances (i.e., different ages, risk factor levels, and lifestyles). This research encompasses individuals between the ages of 30 and 79 in ten geographically defined areas of China, and numerous scientific articles have already been published based on the findings [18]. These include a sex-specific association between tobacco smoking and obesity, an association between self-rated health status and numerous comorbidities, and an association between fresh fruit consumption and all-cause/cause-specific mortality [19,20,21].

Project Baseline by Verily (269 E Grand Ave, South San Francisco, CA 94080, USA) is a newer effort that gathers biometric data, health records, and self-report information, with the goal of creating a “map” of human health [17,22]. This is being done by collecting data from smart devices used by 10,000 people over the span of four years, combined with genetic tests and other data (i.e., participants’ feelings, health records, family histories, periodic lab tests on urine, saliva, and blood); as of 2021, this study has yet to be completed [23]. The goal is to improve overall health and predict when someone might suffer a medical emergency such as a stroke or seizure [16,24]. Additionally, they have smaller studies (i.e., mental health, gut health, heart health) where individuals can participate even if they are not a part of the larger study. Since the start of this venture, they have posted numerous study results on their website’s blog (not peer-reviewed) for the public to view.

Third, a collaboration involving deCODE Genetics (Sturlugata 8, 101 Reykjavik, Iceland) [25] and SomaLogic (2945 Wilderness Pl, Boulder, CO 80301, USA) [26] plans to conduct a large-scale protein analysis of 40,000 human samples [27]. The goal is to gather information that will help to enhance the understanding of how human disease, health, and health outcomes are mediated through proteins. While this collaboration has not been highly publicized since its announcement in 2018, they have since published study results that identified a new canonical protein biomarker associated with osteoporosis in an Icelandic population of ~37,000 individuals [28].

Notably different from the other groups is the Research Institute for Aging (RIA) (250 Laurelwood Dr, Waterloo, ON N2J 0E2, Canada) [29], which is a charitable organization that provides a collaborative space to learn from older adults and their care partners, a training space to educate future healthcare professionals, as well as research labs to train the next generation of experts in aging. Being the first purpose-built teaching long-term care home in Ontario, Canada, the goal is to promote and support the health, well-being, quality of life, and care of seniors by using research findings to create and improve resources, programs, education/training, practices, and policies. Their research agenda revolves around four key programs. The Agri-Food for Healthy Aging (A-HA) program aims to improve the health and well-being of the elderly with the innovative use of food to promote optimal nutrition for older adults and enhance food and mealtime experiences. The GeriMedRisk program is an online and telephone service that connects geriatric specialists with clinicians to help with deprescribing and optimizing medication in both primary and long-term care. The Murray-Alzheimer Research Education Program (MAREP) integrates research and education to help improve dementia care practices. Lastly, the Ontario Centre for Learning, Research and Innovation in Long-Term Care (CLRI) aims at strengthening the quality of care and lifestyle for residents through education, research, and knowledge mobilization.

Other ventures exist to improve research and precision medicine, with the ultimate aim of fully implementing personalized medicine in the future. One of the largest is the All of Us Research Program, by the US National Institutes of Health (NIH) (9000 Rockville Pike, Bethesda, MD 20892, USA). This project, which is part of the Precision Medicine Initiative [30], is collecting data from one million people living in the United States using smart wristbands, sleep sensors, environmental monitors, electronic health records, and physical measurements and is also conducting genetic and microbiome sequencing. Individual differences in lifestyle, environment, and biology will be analyzed in the hopes of building a diverse database that will uncover paths toward delivering precision and personalized medicine [16]. Thus far, they have already recruited 386,000 adults, with 80% from populations previously underrepresented in biomedical research [31].

The HUMAN Project, run by the Kavli Foundation (New York University, 50 West 4th St, New York, NY 10012, USA), seeks to provide insight “on what makes us ill and what makes us well” [32,33]. What makes this venture stand apart from the others is the number of social measurements undertaken, as opposed to largely physical health-related measurements. They propose to evaluate health and psychological well-being, education and employment, social network, finances, and other factors such as criminal justice and civil court interactions, media consumption, religious affiliation, and volunteer activities. The study design is to follow 10,000 people living in New York City for 20 years [34]. Notably, the results of this study will be primarily based on city living, which is very different from living in the suburbs or rurally. However, with 56.2% of the global population and more than 80% of North America’s population now living in large metropolitan areas, the results will be of broad significance [35,36].

Another private venture aiming to create a “map” that shows us our current health status is iCarbonX (4018 Qiaxiang Rd, Hua Qiao Cheng, Nanshan Qu). Their stated goal is to create a future in which people can “enhance their own health and defeat disease” [37]. They sought to investigate more deeply than simply genetics, and thus to pool results from genomics, proteomics, metabolomics, transcriptomics, immune responses, analyses of gut bacteria, and lifestyle factors. Their ultimate goal was to create a mobile phone application with which users are able to access their results and receive advice on nutritional, exercise, and sleep needs, as well as offer a range of personalized products and services that can bring people closer to their health goals [38]. However, since 2019, they have “refocused” on establishing their multi-omics laboratory and data analysis platform. It seems that they are now going to be collaborating with biopharmaceutical and biotechnology companies to develop new diagnostic and pharmaceutical products.

### 2.2. Business Ventures (Paying Clients)

Business ventures differ from those discussed above as they provide scientific wellness and personalized medicine approaches for a fee. Individuals pay to receive a “personalized” treatment plan and lifestyle recommendations for the prevention of disease and maintenance of health. One widely known venture, 23andMe (349 Oyster Point Blvd, South San Francisco, CA 94080, USA), performs genetic analyses (using saliva samples) and provides data ostensibly concerning genetic insight into the ancestry, traits, and health of a client. However, it does not provide recommendations or coaching of any kind. While, in and of itself, this does not seem to contribute to personalized medicine or scientific wellness, they collaborate with numerous universities, industries, and non-profits. These collaborations consist of exchanging de-identified consumer genetic information for profit [39]. Notably, 23andMe has recently announced a merger with the telemedicine platform, Lemonaid Health (870 Market St, Suite 415, San Francisco, CA 94102, USA), and will acquire the company by the end of 2021. The aim of this merger is to provide 23andMe clients with personalized healthcare coaching based on their genes, environment, and lifestyle [40,41].

Human Longevity Inc. (4570 Executive Dr, San Diego, CA 92121, USA) aims to build a comprehensive database of human genotypes and phenotypes that can be subjected to machine learning in order to develop new ways to prevent diseases associated with aging. They also provide a commercial platform (Health Nucleus 100+) designed to assess the current health status and potential health risks of clients [42]. Interested individuals pay and receive a baseline assessment that includes a whole-body MRI, whole-genome sequencing, coronary calcium scoring, body composition, core labs, medical history, and an optional cardiac MRI. They then receive an annual follow-up of every assessment, except coronary calcium scoring, and personalized health reports as long as they continue with their membership. These health reports provide members with personalized and actionable information to optimize their health and prevent disease. Notably, the current total cost for this service is USD 19,800 per client per year for the first year, and then USD 10,000 per year for renewal of membership [43].

Finally, and perhaps most important to ongoing discussions, is Arivale (710 2nd Ave S, #410, Seattle, WA 98104, USA. The founders of this company, Leroy Hood and colleagues, originally conducted a study that collected and analyzed data from the whole-genome sequences, clinical tests, blood biomarkers (via metabolomics and proteomics), and microbiomes of 108 individuals. The goal was to collect multi-“omic” longitudinal data to look for signs of disease prognosis or risk factors in individuals [15]. The success of the pilot study prompted the development of Arivale and the 100K Wellness Project [44]. Participation in Arivale required interested individuals to pay approximately USD 4000 per year to receive lifestyle and wellness recommendations from the Arivale “coaches”, who were registered nurses, dietitians, or certified nutritionists [16,44]. The goal was to create numerous data points for many different diseases and use these data to guide future preventive medicine efforts [44]. Essentially, Arivale was selling scientific wellness in the shape of multi-omics, and molecular tests to highlight different (potential) health concerns to participants. While many individuals were intrigued by this business venture, Arivale was unable to find the right combination of price point, product, and distribution [17]. After only four years, Arivale was forced to shut down as they were unable to recruit enough customers to cover the cost of providing the services.

## 3. Lessons Learned

With the many strategies now implemented in communities, on both a small and larger scale, it is important to critically evaluate the pros and cons. First, any unbiased efforts to broadly improve healthspan are to be applauded. However, to be healthy and to take care of oneself has a price. The business ventures exchanging recommendations and coaching for a fee are, generally, catering to a relatively affluent portion of the population; therefore, the data collected are not necessarily generalizable to the population as a whole [17]. This is where the research-centric ventures are at an advantage as they cover a broader demographic and likely tend to focus on the most practical, proven, and affordable interventions rather than the latest and/or largely untested (e.g., “fad”) approaches garnering attention (and money) from a somewhat poorly informed lay public—and seemingly from (un)informed investors as well. Nonetheless, scientific wellness is not inexpensive, although it need not be a serious financial burden either.

Notably, it could be argued that the direct-to-consumer companies are acting prematurely and that providing genotype-based recommendations may prove to be only as effective as the “one-size-fits-all” recommendations, as that is the nature of their data interpretation. Without detailed and very large-scale research data to support their recommendations, there is no way of knowing how accurate they truly are [45]. While the necessary genomic analyses are becoming more routine with continuous technological developments, considerable reannotation and reinterpretation of data is routine and ongoing, particularly as sample numbers become more appropriately large and rigorous biochemical/physiological measures are implemented to test the theories arising from genomic studies. Simply, linkage of genomic variations to some phenotypic traits does not establish causality and most certainly should not be assumed to. That said, a predictive use is clearly plausible and may be useful in promoting certain lifestyle changes. However, researchers and service providers do not yet appear to fully appreciate the impact of providing a layperson with scientific data reports and leaving interpretation open to them [17,46]. In addition to the obvious issue that a layperson does not have sufficient background knowledge to interpret these reports, there is also the risk of emotional and psychological stress caused by any potentially negative findings. There is thus much to be done on understanding the knowledge translation aspects of such endeavors, whether “coaches” are involved or not. Indeed, there are even data indicating that members of the general population become somewhat complacent about their health when they receive what are interpreted to be “negative” genetic reports, believing that genes are fate and that they need not bother with their health as “there is nothing to be done anyway”. Such a broad lack of appropriate education on these matters, prior to receiving testing and its results, is, at best, negligent, as genes are not fate; research continuously establishes that, even in the case of many monogenic disorders, lifestyle can have the most substantial impact on phenotype and disease progression [47,48,49,50,51]. The majority of scientific wellness ventures are thus admirably geared towards preventing diseases and promoting health by changing lifestyles. However, without detailed scientific knowledge underlying the meaning of much of the current assay data available, it is possible that they might also be causing some degree of harm as well. Furthermore, along with the lifestyle recommendations currently provided by these companies, it is important to note that many individuals are not capable of making extensive changes to their current lifestyle according to what disease(s) they might develop in the future [17]. In the broader social context, early education in and uptake of a healthy lifestyle—rather than attempts to “rejuvenate” and reverse the damage—would be the most beneficial and impactful development in terms of increasing healthspan and reducing long-term healthcare burdens.

From the perspective of assessment, high-quality research requires a substantial investment of time, resources, skilled personnel, and money. The testing suite alone used by Human Longevity Inc., including the full-body MRI, has a cost of USD 25,000 [17]. This being said, it is quite clear why the RIA, rather than Arivale, has been successful. While the goal of the RIA is to change/improve the way we age, and not necessarily with what most would immediately identify as personalized medicine or scientific wellness approaches, it is important to note this organization as their model provides insight concerning how to successfully implement such approaches in a community setting. Their business model puts them in a better position to implement personalized medicine and scientific wellness in the future as the RIA is non-profit and all revenue over cost is fully turned back to supporting further research activities. This permits the RIA to focus predominantly on research quality rather than on the volume of uptake and a corporate profit margin.

It thus seems clear that the main reason that Arivale was unsuccessful was because it could not recruit enough participants of means to cover the costs incurred. Additionally, it is likely that they sought to simultaneously analyze too many data points, at a time when we are only beginning to realize how little we understand about omics data and molecular interactions (possibly a similar reason for iCarbonX’s “refocusing” of their platform). The Arivale goal was to collect data that provided insight into how the body works. Inherently, this requires an understanding of underlying molecular mechanisms and it seems that current research and medicine could not effectively address or utilize these vast quantities of data to provide the promised feedback to clients (nor, likely, have appropriate interventions been developed). In the end, Arivale could not recover from trying to do too much, too soon, and going too big, too fast. This may yet well prove to be an issue for some of the other endeavors. A second advantage seen with the RIA model is that all study participants essentially reside in the research center. It is a challenge to keep people engaged in these types of studies/lifestyle changes over many months or years; having to contact and keep “clients” engaged online or over the phone is only a further complication [16]. However, there is some genuine promise in digital devices (e.g., wearable technology), overcoming this “limitation” of localization.

**Figure 2:**
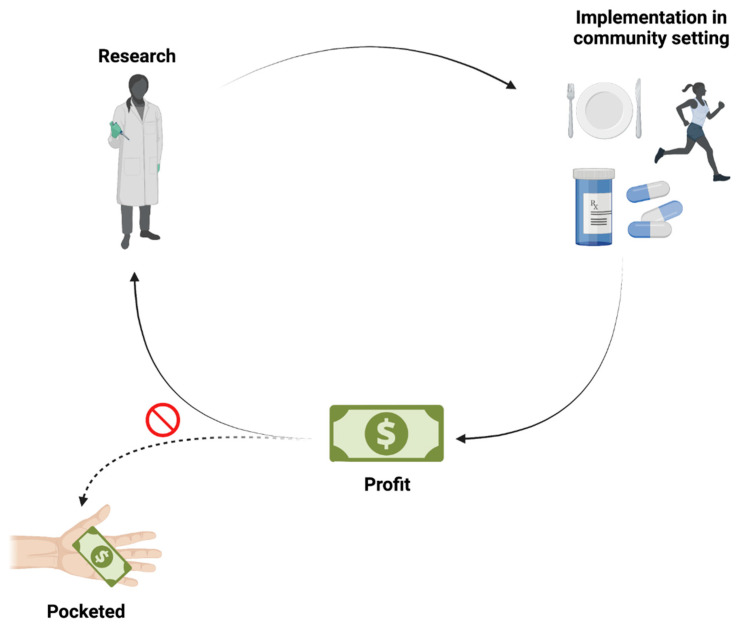
The dilemma. A broadly applicable scientific wellness model (i.e., across healthspan/lifespan) that balances both profit and a focus on generating high-quality research has yet to be implemented in the community. Striking such a balance would seem to be the necessary target in terms of affordable scientific wellness based on rigorous, quantitative research. As depicted in the figure, when profit is only partially reinvested into the research, quantitative science and effective implementation in the community are at risk.

Thus, to successfully implement scientific wellness in both local and broader community settings, it currently seems most advantageous to do so using a research model as opposed to a business model. While scientific wellness is complex and not cheap, successfully establishing this type of endeavor in a community cannot be done successfully when focused primarily on making a profit. Unfortunately, at this moment in history, it seems clear that we cannot have one without sacrificing the other (Figure 2).

## 4. Conclusions

At present, the research needed to fully implement scientific wellness in a public setting is lacking. While scientific wellness may well be the future of healthcare and could substantially reduce healthcare burdens/costs while maximizing healthspan, currently, there is insufficient emphasis on quality research [14,52] and perhaps too much emphasis on potentially exploitive economic opportunities. It seems that many of the individuals or companies advertising and promoting scientific wellness are suffering from Shiny Object Syndrome (SOS)—a term often used in the marketing industry—and while scientific wellness is a healthcare-related concept, SOS is, regrettably, also quite a fitting description of some of what is being marketed. This syndrome occurs when a new (and generally untested) idea captures an individual’s attention so fully that they become distracted from the big picture, causing them to neglect what is really important (e.g., genuine facts, evidence, data, practicality, and fulfilment of or firm establishment of critical criteria) [53,54]. Scientific wellness involves numerous interconnected mechanisms that cannot be isolated from one another [5,6]. Thus, a systems (biology) approach is needed in evaluating the available data and necessary experimental designs to address targeted questions. In order to modify healthcare to be more personal and to identify underlying molecular mechanisms, accepting, embracing, and addressing this complexity is necessary. As the development of Western evidence-based medicine took generations of research (and that of Eastern traditions has a background of millennia), it could be concluded that attempting to rush scientific wellness into large-scale practice before we have a deeper and more systematic understanding of underlying molecular mechanisms and our analytical tools may prove ineffective, if not even detrimental. Many of the ongoing research ventures described above are in early stages and will take years to complete data collection and detailed analysis. Therefore, until the research is completed, one might argue that we should err on the side of caution and not jump ahead of the science. It will be those who take the long and difficult road, being cautious and rigorous, that will emerge to see the benefits on the other side, leaving those with SOS by the wayside, or worse. The focus must be first and foremost on healthspan. Thus, we believe that research-based ventures, such as the RIA model (but introducing more broadly applicable scales and targets), seem less a risk than the for-profit ventures described above and tend to avoid issues of “wealth-and-health”.

Additionally, companies selling or otherwise sharing genetic information introduce additional (health) risks for clients already paying substantial fees. While the anonymized data are currently private, it may be possible in the future for the data to be linked to the individual. Genetic information can never truly be de-identified as it is unique to each individual and what is now considered private could one day become public [55,56]. While the contribution of genetic samples to research has provided a wealth of knowledge, many precautions need to be taken to ensure that the data cannot be tracked back to the individual. This is not only relevant to the individual sharing their data but also to their relatives. One database, GEDmatch (11111 Flintkote Ave, San Diego, CA 92121, USA), allows individuals to upload their DNA to a public server [57]. The goal of the website is to create family trees and find other individuals (i.e., relatives) with similar genetics. The drawback is that there are no systems in place to protect the relatives of these individuals and, thus, they can be identified without their consent or genetic material [58,59]. Almost all of the ventures mentioned above present significant challenges in terms of the privacy of genomic and biomedical data. While there is the concern of identifying individuals without their consent, information about genetic conditions and predispositions poses a major threat if employment and insurance agencies are able to obtain these records and allow the data to affect the individual’s opportunities in the future [60].

Finally, it is not as though there are no data concerning scientific wellness and thus proven long-term strategies for generally improving and/or maintaining health and wellness. These can be as simple (and financially relatively reasonable) as a balanced diet emphasizing moderation, regular reasonably vigorous exercise, continuous learning, a personally satisfying level of interpersonal/community interactions, and an effective means of de-stressing [50,61,62,63,64]. These are not “sexy” and do not involve the latest technological wonder devices or “brought-back-from-the-end-of-the-world” botanical compound, but they are tried and tested. As for the latest and greatest isolated nutrient or health-promoting machine, some will stand the test of time but many will (hopefully quickly) go the way of radium water and similar health fads [65,66]. This is not meant in any way to downplay the importance of scientific wellness, precision, or personalized medicine but rather to urge genuine scientific/evidence-based practice in their development and rational deployment. This demands a deeper and more critical understanding of the assays and associated analytical methods employed, as well as educating the public as to what the data actually indicate, as opposed to how some would like to interpret the data. In the end, it must be about quality healthcare, not the building of scientific/medical/business empires (on foundations of quicksand). Only the most solid of foundations stand the tests of time.
